# Parental Separation and School Performance Among Children of Immigrant Mothers in Sweden

**DOI:** 10.1007/s10680-017-9419-3

**Published:** 2017-03-22

**Authors:** Jeylan Erman, Juho Härkönen

**Affiliations:** 1 Department of Sociology, University of Pennsylvania, 239 McNeil Building, 3718 Locus Walk, Philadelphia, PA 19104-6299 USA; 2 Department of Sociology, Stockholm University, 106 91 Stockholm, Sweden

**Keywords:** Divorce, Separation, Race and ethnicity, Education, Inequality

## Abstract

Immigration and family change are two demographic processes that have changed the face of European societies and are associated with inequalities in child outcomes. Yet there is little research outside the USA on whether the effects of family dynamics on children’s life chances vary by immigrant background. We asked whether the effect of parental separation on educational achievement varies between immigrant backgrounds (ancestries) in Sweden. We used Swedish population register data on two birth cohorts (born in 1995 and 1996) of Swedish-born children and analyzed parental separation penalties on grade sums and non-passing grades (measured at ninth grade) across ten ancestry groups, defined by the mother’s country of birth. We found that the parental separation effects vary across ancestries, being weakest among children with Chilean-born mothers and strongest among children with mothers born in Bosnia and Herzegovina. In general, the effects were weaker in groups in which parental separation was a more common experience.

## Introduction

Family change and immigration are among the demographic processes that have changed the face of European societies. Both are also associated with inequalities in children’s life chances, and large literatures have investigated differences in educational and other socioeconomic outcomes across family structures (McLanahan and Sandefur 1994; Amato 2000; Härkönen 2014) and by immigrant status (Heath et al. 2008). How are these outcomes shaped by the intersection of these two, do effects of family structures and dynamics vary by immigrant background? Most research on this question comes from the USA and finds that parental divorce effects can indeed vary between racial and ethnic groups (e.g., Heard 2007; McLanahan and Sandefur 1994; McLoyd et al. 2000; Sun and Li 2007).

Do these patterns generalize to Europe, where the histories of immigration and racial/ethnic relations are quite different? Two studies, one from the Netherlands (Kalmijn 2010) and another comparing four countries (Kalmijn forthcoming), suggest so. The former study included only one immigrant group (Caribbeans) and the latter largely resorted to large groupings of origin countries. While these studies provide evidence for the above question, additional research analyzing multiple well-defined immigrant groups is warranted for further evidence and for contributing to the theoretical understanding of variation in parental separation effects.

This study contributes to the emerging European literature on the interactive effects of immigrant background—or, ancestry—and school performance by analyzing this question in Sweden. Sweden has a large immigrant population, where 16% of the population in 2014 was born abroad and another 5% had two foreign-born parents (Statistics Sweden 2015). Sweden’s immigrant population is also very diverse and includes large immigrant populations that have immigrated at different times from the other Nordic countries and the rest of Europe, Middle East, Africa, and South America. In comparison with most surveys, Sweden’s large population register data enable more precise identification of countries of origin and, thus, more detailed analyses of ancestry group variation. Furthermore, in contrast to cross-nationally comparative work, comparison of groups in a single country allows for variation in the socioeconomic position, social cohesion, and cultural practices between the different groups, while holding constant the country’s broad institutional and social context. We analyze how the relationship between parental separation and school performance varies between the groups, how much of this variation is due to group differences in demographic and socioeconomic factors, and whether the remaining variation is systematically related to the prevalence of having separated parents and to differences in social support. Our analysis thus contributes to understanding differences in family structure effects between ancestry groups in particular and heterogeneous consequences of parental separation in general (Amato 2010). Finally, our analyses further our understanding of the factors that shape the life chances among children of immigrants.

Specifically, we analyze the relationship between parental separation and school performance among Swedish-born children across ten ancestry groups: those whose mother was born in Sweden, Chile, Finland, East Africa (Djibouti, Eritrea, Somalia, Sudan, and Ethiopia), Iran, Iraq, Poland, Bosnia, and Herzegovina, the rest of the former Yugoslavia (Croatia, Macedonia, Montenegro, Serbia, and Slovenia), or Turkey. These are among the largest immigrant groups in Sweden and represent Sweden’s immigrant population’s diversity with respect to migration history, cultural distance, and integration. This diversity is also visible in the school performance of children of these immigrants, with those of Iranian and East European descent performing better than native Swedes, whereas children of immigrants from the Nordic countries, the Middle East, and Africa show poorer school performance (Jonsson and Rudolphi 2011).

We use population register data of 152,186 children born in Sweden in 1995 and 1996 from the Sweden in Time—Activities and Relationships (STAR) data files, which are compiled by Statistics Sweden for Stockholm University. We analyze the association between parental separation and school grades [measured as grade sums and incomplete grades (cf. Jonsson and Rudolphi 2011)] across the ten ancestry groups, defined by the mother’s country of birth. The focus on children born in Sweden enables us to measure the children’s family histories from the register data we use.

## Background

### Parental Separation and Educational Performance

A large literature has documented how parental separation is associated with poorer filial outcomes in terms of psychological well-being, scholastic achievement, social relationships, and adult socioeconomic status, and how these associations can be found for a broad range of societies (Amato 2000, 2010; Amato and James 2010; Härkönen 2014).

Because educational achievement is a strong predictor of later life outcomes—whether socioeconomic or otherwise—much of the literature on parental separation effects has considered whether family dissolution disrupts educational outcomes (e.g., Amato 2001; Cherlin et al. 1991; Frisco et al. 2007; Grätz 2015; Jonsson and Gähler 1997; Bernardi and Radl 2014). The conclusion from this literature is that an experience of parental separation is associated with poorer educational outcomes, whether measured as GPAs, standardized tests, educational transitions, or highest attained education.

The correlations between parental separation and educational outcomes are partly due to selection (Amato 2000, 2010; Härkönen 2014; Härkönen et al. 2017, this issue). In many countries, separating couples are less educated than those who do not separate (Härkönen and Dronkers 2006) and they also differ on many other characteristics—such as levels of conflict—which may predict lower educational performance. Nevertheless, many findings indicate that parental separation can have a negative causal effect on education, even though these effects are substantially weaker than the crude associations (Amato 2010; McLanahan et al. 2013).

The negative effects of parental separation on educational outcomes have been explained by socioeconomic, psychological, and social pathways. Separation often leads to a loss in socioeconomic resources, and it is an important predictor of transitions into poverty, particularly for women (DiPrete and McManus 2000; Uunk 2004; Callens and Croux 2009). Loss of socioeconomic resources and economic disadvantage explain part of the parental separation penalty on educational outcomes (McLanahan and Sandefur 1994; Thomson et al. 1994; Jonsson and Gähler 1997; Bernardi and Boertien 2017, this issue). Parental separation often also means residential mobility, potentially because of the above-mentioned socioeconomic consequences, which can destabilize children’s social networks and other aspects of their life (McLanahan and Sandefur 1994; Amato 2000).

Parental separation can have negative psychological effects in the short and the long run, ranging from feelings of sadness and loss to clinical psychiatric conditions, such as depression (Amato 2000; Amato and James 2010), which can translate into poorer educational outcomes. Parents going through their own emotional work may have a reduced capability to exert control over their children and may engage in uncoordinated parenting (Amato 1993). Family disruption may also lead to a deterioration in the relationship between the child and the parent—especially the father—weakening access to that parent’s resources and help (Albertini and Garriga 2011; Aquilino 1994).

Parental separation can also be the initial trigger to additional family transitions, such as step-family formation, and their potential dissolutions. These can further increase children’s family life instability, which may in itself have negative consequences on children’s well-being and educational performance (Amato 2010; Fomby and Cherlin 2007).

The associations and effects reviewed above hold on average, but all children do not suffer from their parents’ separation; for the majority, any (long-term) effects are nil or minor, and some children benefit from exiting a dysfunctional family (Dronkers 1999; Amato and Anthony 2014). Yet, other children experience long-lasting negative consequences. Recent research has paid increasing attention to the factors that create vulnerability and resilience in the face of parental separations (Amato 2010; Amato and Anthony 2014; Bernardi and Radl 2014; Grätz 2015). Ancestral, ethnic, or racial background is one potential modifier of the association between parental separation and child outcomes, and we turn next to reviewing the relevant literature.

### Heterogeneous Effects by Ancestry

Most research on heterogeneity in parental separation effects by ethnic, racial, and ancestral background comes from the USA. Several studies have reported that parental separation has weaker effects—on educational outcomes, psychological adjustment, and family demographic behaviors—for Black than for White Americans (McLanahan and Bumpass 1988; Amato and Keith 1991; McLanahan and Sandefur 1994; Smith 1997; Wu and Thomson 2001; Heard 2007; Fomby and Cherlin 2007; Lee and McLanahan 2015; for a study finding no differences, Sun and Li 2007) and other studies have extended this comparison to Hispanics and Asians (Sun and Li 2007). In a European context, Kalmijn (2010, forthcoming) showed that the associations between parental separation and father absence and various aspects of well-being and demographic behaviors can vary between ancestries. For example, a study on England, Germany, the Netherlands, and Sweden revealed that father absence is not associated with self-esteem among children with Caribbean or African ancestry, whereas father absence is related to lower self-esteem among natives and even more so among children with Middle Eastern and South-Central Asian ancestries (Kalmijn forthcoming).

These results lead us to expect variation in the association between parental separation and school performance in Sweden as well. Below, we group the discussion of the potential reasons for this variation into three sets of explanations: compositional differences and selection, effects of separation on socioeconomic outcomes, and differences in the remaining penalty due to the incidence rate of separation, and in social support.

#### Selection: Heterogeneous Associations Due to Compositional Differences

Immigrants and other minority groups often have fewer socioeconomic resources, such as lower education, weaker labor market performance, and less incomes. These can shape the association between parental separation and school performance. Low education increases the risk of separation in Sweden as well as in many other countries (Härkönen and Dronkers 2006), and the group differences in educational attainment can consequently account for part of the variation in the association between parental separation and school performance. Furthermore, it is possible—although not known empirically—that education shapes separation risks differently depending on the ancestry group, which would imply that parental education confounds the association between parental separation and school performance differently.

Other confounders that can shape group differences in the parental separation—school performance relationship—include young parental age, which increases separation risks (Lyngstad and Jalovaara 2010) and can have negative effects on educational outcomes (Kalmijn and Kraaykamp 2005), number of siblings, which also predicts separation risk and school performance (Lyngstad and Jalovaara 2010; Steelman et al. 2002), and having a parent from the native population (Smith et al. 2012). Women from all of the immigrant groups considered here have lower rates of marriage to Swedish men than native Swedish women do. It has, however, been most common among Finnish and Polish women and least common among women from Iraq and Bosnia-Herzegovina (Dribe and Lundh 2008).

Among immigrants, the association between parental separation and school performance can be further shaped by the time the immigrant parents have spent in the host country and the reason for their immigration. Those who have spent longer time in the host country can be more socialized into the family behaviors and culture of the host country and the association between parental separation and school performance can thus be more similar to the native group. Those who arrived as labor migrants can also be more integrated, at least socioeconomically, to the host country. In addition, migration itself—especially when involuntary—can be a taxing process and the stressors related to it and difficulties in adapting to the new environment can increase the likelihood of conflict and separation (Lyngstad and Jalovaara 2010) as well as have independent effects on children’s outcomes. Consequently, the association between parental separation and school performance can be expected to be more similar among groups that have been in Sweden for a longer time. Parental separation effects can also differ between groups that predominantly arrived as labor migrants compared to those who arrived predominantly as refugees or for other reasons.

#### Variation Due to Socioeconomic Consequences of Parental Separation

The economic consequences of separation can also vary between ancestry groups. Differences in economic consequences have been pointed out as an explanation for differences in parental separation effects between Blacks and Whites in the USA (e.g., McLanahan and Sandefur 1994; Smith 1997; Sun and Li 2007). In Sweden, foreign-born individuals have generally fared worse in terms of employment and income than native-born individuals, and refugees and those from outside Western Europe have had the poorest outcomes (Lemaître 2007; Shroder 2007). Among all groups, foreign-born women are the worst-off and can face discrimination as a result of being both an immigrant and female (Shroder 2007). They can also be more vulnerable to the economic consequences of separation. Alternatively, they may face fewer losses due to family dissolution if their economic condition was weak to begin with (cf. Sun and Li 2007).

#### Variation Due to Differences in Separation Incidence

Many studies have found differences between ethnic, racial, and ancestral groups in the association between parental separation and child outcomes even after controlling for socio-demographic variables. A common explanation for differences in these residual associations refers to the incidence and acceptance of family dissolutions in the different groups (Amato and Keith 1991; McLanahan and Sandefur 1994; Sun and Li 2007; Kalmijn 2010, forthcoming). American research has consistently shown that Black Americans have the highest family dissolution rates of the groups considered (Amato 2010). Group differences have also been found in Europe (Kalmijn 2010; Hannemann and Kulu 2016). In Sweden, of the ancestries considered here, divorce rates are the lowest among native Swedes and Turkish immigrants (28% divorced 15 years after the wedding) and most common among immigrants from the Horn of Africa (58%) (Andersson et al. 2015). Worth mentioning is that the divorce rates of immigrant groups are generally higher than those of native Swedes, even though crude divorce rates in the countries of origin are often considerably lower. For example, the United Nations (2009, 2013) reported that the crude divorce rate around 2000–2005 was above 2 in Sweden and Finland, 0.5 or below in Bosnia-Herzegovina and Chile, and between 1 and 2 in the other countries of origin.

The general argument behind the “incidence and acceptance” explanation is that in groups with higher rates of family dissolution, parental separations are less stigmatized and single motherhood is a more institutionalized living arrangement, which leads to lower psychological distress and better coping mechanisms in the face of family dissolution. This may then translate into weaker effects of parental separation on school performance (Amato and Keith 1991; Kalmijn forthcoming). The relevance of this argument can be questioned in a country such as Sweden, in which parental separation is common and its disapproval is low (Rijken and Liefbroer 2012). It has also been questioned in some cross-national research. For example, Pong et al. (2003) found that the single- and two-parent achievement gap was larger in countries where single parenthood is more prevalent (cf. also Kreidl et al. 2017). Any association between the incidence of parental separation and parental separation penalties may thus, alternatively, reflect differences in the selectivity of dissolved families in terms of predictors of negative educational outcomes. The family dissolution process as well as thresholds to separating (for example, by the level of conflict) can differ between groups in which separations are more/less common. Consequently, separated families in these groups can differ according to characteristics, which are related to children’s school performance.

#### Variation Due to Differences in Social Support

Heterogeneous parental separation effects may also reflect group differences in social support and social networks. This argument has been prevalent in explaining the weaker family disruption effects among Black Americans and points to a stronger importance of kin and other social networks outside the nuclear family (Hunter 1997; Smith 1997; McLoyd et al. 2000). In times of crisis—such as economic troubles or family disruption—these social networks help buffer the potentially adverse effects. In particular, groups may vary in how much grandmothers and other female kin provide help, either through intergenerational co-residence or otherwise (Haxton and Harknett 2009; Hunter 1997; Schans and Komter 2010). In the case of immigrants, access to support from kin especially can be limited by the fact that many immigrants’ kin do not live in the host country. Likewise, research has found that immigrants often have limited access to social capital, but when their lower socioeconomic status is accounted for (Verhaeghe et al. 2015; van Tubergen and Volker 2015) or when studying the children of immigrants (Behtoui and Neergaard 2015), this disadvantage often disappears or even reverses. Some immigrant groups may thus be better willing or equipped to provide support in the face of family dissolution, conditional on having kin in the host country who can provide it.

### Immigrant Groups in Sweden

Sweden’s immigration population is diverse and shaped by multiple waves of immigration.

Labor migration from Finland was prominent especially between the late 1960s and early 1980s (Andersson et al. 2015; Korkiasaari and Söderling 2003). Iranians, Chileans, Yugoslavians, and Poles have likewise a relatively long history in Sweden. The 1980s saw an influx of, often well-educated, Iranian (Darvishpour 1999) and Polish political refugees, although many Polish women also arrived to partner a Swedish man (Andersson et al. 2015). Many Chilean political refugees arrived in the wake of the dictatorial regime in the 1970s, followed by economic migration from Chile in the 1980s (Cronemo 2012). Migration from the former Yugoslavia consisted of (in particular, Serbian and Croatian) labor migrants starting in the 1960s and later, in the 1990s, of refugees fleeing the Balkan wars, particularly from Bosnia and Herzegovina. A similar pattern existed among immigrants from Turkey, the first of whom arrived mainly as labor migrants (often from a single Anatolian city, Kulu) but were later increasingly refugees, particularly from the Kurdish minority. Many immigrants from East Africa and Iraq came to Sweden as refugees, the first consisting especially of Somalis who began arriving in the 1990s, whereas immigration from Iraq began in the 1980s and increased since 2003 (Andersson et al. 2015).

The above description of the immigrant groups considered in this study highlights the history and some of the characteristics of the different immigration waves to Sweden. The characteristics of migrants can shape the associations between parental separation and school performance in these groups, both by affecting the socio-demographic compositions of these groups (confounding effects) as well as the consequences of the separations (mediators). The description also underlines that immigrants do not constitute a cross section of the sending country’s population, nor are they homogeneous in terms of ethnicity. Furthermore, due to selective return and onward migration (Nekby 2006), the characteristics of immigrants who stayed in Sweden are not necessarily the same as of those who arrived there in the first place. These processes shape the profile of the immigrant populations and potentially the experiences of (parental) separation among them. Another implication of these processes is that because the characteristics of immigrant groups from one country of origin can vary in different countries of destination, our findings on parental separation effects for a specific immigrant group do not necessarily generalize to other receiving countries.

### The Present Study

We compare gaps in school grades between adolescents whose parents separated and those whose parents remained together (here referred to as the parental separation penalty) in and across ten ancestry groups, defined by the mother’s country of birth. The mother’s country of birth was chosen to define ancestry groups with the assumption that most children reside either exclusively or to a large extent with their mothers after a parental separation. Given the heterogeneity in backgrounds within regions and even within countries of origin, the ten ancestral groups chosen here do not necessarily overlap with recognized and self-identified ethnic groups. However, they are arguably more internally homogenous than many alternative classifications, such as groupings based on geography and economic development.

The purpose of this study is threefold. First, we describe differences in the parental separation gap in grades in our ten ancestry groups. Second, we analyze to what extent these gaps and the differences in them are due to socioeconomic and demographic differences between dissolved and intact families. We group the socioeconomic and demographic variables into those, which are primarily determined before parental separation (and thus act as confounding factors and reflect the socio-demographic composition of dissolved families), and those, which can also be affected by the parental separation (which can act as mediating variables). Third, we assess whether the differences in these gaps (net of socioeconomic and demographic variables) relate to the incidence of parental separation as well as the prevalence of three-generation households. The former relates to the “incidence and acceptance” argument outlined above. The latter is used to proxy access to kin support among the different groups. The prevalence of three-generation households is a measure related to the degree of available intergenerational support (e.g., Reher 1998), and although this measure is not the only one indicating intergenerational support, it is available at the ancestry group level, unlike many other potential measures. It is also closely related to the arguments of the importance of grandparental support, prevalent in the American literature on racial differences in family dissolution effects.

We formulate the following hypotheses:

#### **Hypothesis 1**

The association between parental separation and school performance varies across ancestry groups.

#### **Hypothesis 2**

This variation can be explained by socioeconomic and demographic differences between the ancestry groups.

#### **Hypothesis 3**

The parental separation penalty—i.e., the association net of socioeconomic and demographic composition—is smaller in groups with higher parental separation incidence.

#### **Hypothesis 4**

The parental separation penalty is smaller in groups with higher prevalence of three-generational families.

## Data and Methods

We used data for children born in Sweden in 1995 and 1996 from various population registries annually collected and maintained by Swedish authorities, including tax and school registries and the LISA database. These registries provide information on all individuals in Sweden, including place of residence, country of birth, immigration history, income and school performance, as well as other basic socioeconomic information. Information on households can also be constructed from these data. The registries are thus fitting for a study on ancestral background, parental separation, and children’s educational attainment. During the time of this writing, the register data coverage extended to 2012. We focused on children born in 1995 and 1996 and their school grades the year they turned 16 in 2011 and 2012 (at the end of the ninth grade of comprehensive school), respectively. This restriction enables us to include a wide portrait of Sweden’s immigrant population, many of whom arrived in the 1990s. Ten ancestral groups (Sweden, Bosnia-Herzegovina, Chile, East Africa, Finland, Iran, Iraq, Poland, Former Yugoslavia (excluding Bosnia-Herzegovina after the breakup), and Turkey), defined by the mother’s country of birth, were included in the analysis. We restricted our data to children born in Sweden—and not, for example, to children who moved to Sweden themselves—because this way we could reconstruct the children’s family histories. As a robustness check, we ran the analyses using the father’s country of birth as the grouping variable, but this did not change our conclusions. The total number of children born in 1995 and 1996 in these groups was 182,421.

We made additional exclusions to form our analytical sample. Because our study is about parental separation, we, first, excluded 16,313 (8.9%) children who were born to single mothers, i.e., whose parents were not registered in the same address at the end of the child’s birth year. The prevalence of children born to single mothers varied rather markedly, from 30 and 26% among East Africans and Chileans, respectively, to 13–14% among Finns and Poles, and below 10% among the other groups (7.5% among Swedes). As a robustness check, we included these children together with those with separated parents to form a measure of non-intact families, but this did not change our conclusions. However, interestingly, the crude differences in grades among children born to single mothers, children with separated parents, and children with intact families varied, with those born to single mothers having the lowest grades among Swedes, Finns, Poles, and Iranians, whereas the grades were the lowest among children of separated parents in the other groups. A closer analysis of these differences is warranted in future research. Furthermore, we excluded children who died or whose parents died (*n* = 3717) and those whose grades were missing (*n* = 10,205), either because of grade retention or for other reasons, such as emigration. This may affect our results if the missing grades are strongly related to parental separation and differently so between the groups. The total number of observations after these exclusions was 152,186. For the regression analyses, we also dropped the small share of cases with missing information on the independent variables.

### Variables

We used two dependent variables, both of which come from the school grade registries (cf. Jonsson and Rudolphi 2011). The first dependent variable is the *grade sum* (*meritvärde*), which is the sum of the 16 best grades (out of around 20). Each subject is assigned fail (0 points), pass (10), pass with distinction (15), and pass with special distinction (20) and the grade sum thus ranges from 0 to 320. The second dependent variable is a dummy, which measures whether the student got *incomplete grades* from one or more of the core subjects Swedish (or Swedish as second language), English, or Mathematics. The former measure can be seen as a general assessment of scholastic performance and affects the study path a student can attend after comprehensive school. The latter is a measure of failure in academic performance and also indicates inability to enroll in an ordinary upper secondary school program, forcing the student either to leave school or attend preparatory courses.

Parental separation is our main independent variable. In the analysis, separation of parents includes parents in both marriages and co-residential partnerships. Cohabitating couples have been difficult to identify from registers, but residential property-based measures of cohabitation have been used effectively to identify cohabiting couples with children (Thomson and Eriksson 2013). In the population registries, each parent’s residential property at the end of the calendar year is identified. If parents were living in the same property at the year of the child’s birth, they are considered to be in a union. If the parents were in a union at the child’s birth and are no longer living together the year before the grades were measured (when the child was 15 years old), they are considered to have separated. Some parents (*n* = 3729) were not registered in the same residential property at the end of the child’s birth year, but were registered in it at the end of the next year. Robustness checks in which these parents were considered living together when the child was born resulted in very similar results as those reported below. We therefore chose to use the above-described measure, which has been used and validated in previous research (Thomson and Eriksson 2013).

The control variables include the education of both of the parents, the mother’s age at birth (linear and squared), the number of siblings, birth order of the child, sex of the child, whether the father was born in Sweden, birth cohort, municipality at birth, and time since arrival to Sweden. Parental education variables measure the parent’s highest attained education the year before the grades were set (when the child was 15) and are categorized into compulsory (or missing) level, short upper secondary (typically 2 years after the compulsory), long upper secondary (typically 3 years after compulsory), short post-secondary, and tertiary education (university degree or higher). The mother’s age at birth, birth order, and the number of siblings are all predictors of educational achievement and correlate with the probability and age of experiencing parental separation; larger families and those formed at older ages are generally more stable and younger siblings are more likely to experience their parents’ separation (by a specific age) than older siblings. We included a dummy of whether the father was born in Sweden. Having a Swedish father can help in transmitting any educational (dis)advantages of the native population as well as shape the effects of parental separation. We controlled for birth year (whether the child was born in 1995 or 1996). The municipality at year of birth was grouped into four categories: Stockholm, Gothenburg, and Malmö, other towns with 100,000+ inhabitants, towns with 50,000–99,999 inhabitants, and smaller localities. Time since arrival of the foreign-born parent to Sweden was classified as Swedish-born or arrived at least 16 years before the birth of the child (reference), or arrived 11–15 years, 6–10 years, or 0–5 years before the birth of the child.

We also included two mediating variables, measured the year before the grades were set, namely mother’s employment status (employed vs. not employed) and the household’s logged disposable income, adjusted with Sweden’s official equivalence scale. These variables can be affected by the parental separation and can thus mediate any effect of parental separation on school grades. Information on the independent variables is shown in Table 1, and variation by ancestry is shown in Table 2.

**Table 1:** Descriptive statistics of the data

Categorical variables	%	*N*
No incomplete grades	92.1	140,114
At least one incomplete grade	7.9	12,072
Parents separated	31.6	48,109
Parents not separated	68.4	104,777
Mother’s country of birth		
Sweden	91.3	138,959
Bosnia-Herzegovina	1.1	1704
Chile	0.4	587
East Africa	0.8	1141
Finland	1.8	2783
Iran	0.7	1088
Iraq	0.9	1294
Poland	0.5	826
Turkey	1.0	1487
Yugoslavia	1.5	2317
Father born in Sweden	87.9	133,680
Father born abroad	12.1	18,460
Female	48.9	74,451
Male	51.1	77,735
Born in 1995	52.5	79,961
Born in 1996	47.5	72,225
Birth place		
Stockholm, Gothenburg, Malmö	15.0	22,836
100,000+ inhabitants	12.1	18,456
50,000–99,999 inhabitants	22.1	33,632
<50,000 inhabitants	50.1	76,186
Mother’s education		
Compulsory or missing	7.2	10,890
Short secondary	29.4	44,727
Secondary	21.1	32,106
Lower tertiary	18.2	27,666
Tertiary	24.2	36,797
Father’s education		
Compulsory or missing	13.2	20,066
Short secondary	40.9	62,260
Secondary	13.4	20,447
Lower tertiary	15.6	23,661
Tertiary	16.9	25,752
Mother not employed	6.6	9987
Mother employed	93.4	142,198

Continuous variables	Mean	SD
Grade sum	216.0	60.8
Age of the mother at birth	29.4	4.8
Year of arrival (immigrant mums)	1986	9.4
*N* siblings	1.6	1.1
Birth order	1.9	1.0
Logged disposable income	7.6	0.5

Total *N* = 152,186

**Table 2:** Group characteristics by the background variables (mean/%)

	BIH	CHL	E-Afr.	FIN	IRN	IRQ	POL	SWE	TUR	YUG
Age mother	27.9	29.1	28.1	31.4	30.6	28.6	30.2	29.4	27.5	28.5
Post sec., mother	24.2	21.5	11.5	40.3	45.7	37.5	41.2	42.7	10.0	21.7
Post sec., father	24.2	20.9	23.0	29.1	41.6	37.9	28.8	32.0	12.8	24.4
Foreign father	98.8	69.3	96.1	29.8	93.8	99.5	52.6	6.8	94.4	89.6
Employed mother	86.1	85.8	71.4	89.1	83.2	70.5	83.1	93.8	73.7	78.6
Big town/city	23.9	38.0	61.7	29.0	47.5	53.2	40.7	26.8	48.7	35.6
Year of arrival	1993	1986	1991	1975	1990	1992	1988	–	1986	1989
Mother’s N kids	2.4	2.9	4.6	2.8	2.3	3.3	2.3	2.6	3.3	3.1
Birth order	1.8	2.1	2.5	2.2	1.8	2.0	1.8	1.9	2.3	2.4
Ln hh. income	7.49	7.39	7.13	7.56	7.44	7.2	7.42	7.61	7.25	7.38

Post sec.: post-secondary education (short post-secondary or tertiary); big town/city (100,000+ inhabitants, or Stockholm, Gothenburg, Malmö); country/region codes: Bosnia-Herzegovina (BIH), Chile (CHL), East Africa (E-Afr.), Finland (FIN), Iran (IRN), Iraq (IRQ), Poland (POL), Sweden (SWE), Turkey (TUR), Yugoslavia (YUG)

### Methods

The analysis was done in four stages. First, we described the prevalence of having experienced parental separation by age 15 in the ten ancestry groups as well as the parental separation gaps in grade sums and incomplete grades.

Second, we estimated a series of regression models to analyze how much of the parental separation gaps *within* each group can be explained by the control and mediating variables. In other words, these regression models were run separately for each group. The first regression model includes the socio-demographic confounding variables, whereas the second adds maternal employment and logged disposable incomes as the mediating variables. The dependent variable *grade sum* was analyzed using ordinary least squares (OLS) regression. The dependent variable *incomplete grades* was analyzed using linear probability models (LPMs). Because of scaling effects, comparing logistic regression coefficients between models is problematic, whereas LPMs do not have such restrictions (Mood 2010) and are thus more suitable for analyzing how much of the parental separation penalties can be explained by the control and the mediating variables in each group. Robust standard errors were estimated.

Third, we estimated another series of regression models to analyze how big the parental separation penalties between the groups are when conditioning on the control and mediating variables. These between-group differences in parental separation penalties were analyzed with full interaction models, that is, models that interact mother’s country/region of birth with all the independent variables (except the intermediate time since immigration to Sweden, which does not vary among Swedish-born mothers). These models are akin to comparing the estimates from models run separately between the groups. We estimated an empty model without additional control variables, a model with the control variables added, and a model that also adds the mediating variables. The dependent variable *grade sum* was again analyzed using OLS regression, but the dependent variable *incomplete grades* was here analyzed using logistic regressions. This was done because the baseline probability for having at least one incomplete grade varies remarkably between the groups (Table 3). A small percentage point difference in having incomplete grades can mean a big relative difference if the baseline probability is small and vice versa. The logistic regression interaction model presents the between-group differences in the parental separation gap in relative terms (Buis 2010).

**Table 3:** Grade sums means and share (%) receiving one of more incomplete grades, by parental separation and ancestry

	Grade sum			One or more incomplete grades			
	Intact family	Parents separated	*Δ*	Intact family	Parents separated	*Δ*	OR
Bosnia-Herzegovina	221.9	189.2	−32.7	6.6	19.0	12.4	3.3
Chile	196.6	179.9	−16.7	17.9	21.5	3.6	1.3
East Africa	210.0	193.9	−16.1	12.0	19.2	7.2	1.7
Finland	220.9	194.0	−26.9	7.7	13.7	6.0	1.9
Iran	234.4	211.5	−22.9	5.2	12.4	7.2	2.6
Iraq	208.0	187.1	−20.9	13.4	19.6	6.2	1.6
Poland	226.9	198.3	−28.6	6.0	14.1	8.1	2.6
Sweden	225.3	198.5	−26.8	5.5	12.0	6.5	2.3
Turkey	202.3	183.9	−18.4	15.1	22.2	7.1	1.6
Yugoslavia	206.4	178.7	−27.7	12.8	24.3	11.5	2.2

Fourth and finally, we correlated the net (of all the socioeconomic and demographic variables) parental separation penalties in grade sums from each group with two aggregate-level measures at the ancestry group level: the percentage of adolescents in ninth grade who had experienced parental separation, and the percentage of adolescents who live in a three-generational household, conditional on a grandparent identified as living in Sweden. We chose to measure intergenerational support at the aggregate rather than the household level. Residing in three-generation households may be affected by unmeasured (dis)advantages related to school grades at the individual and household level. On the other hand, the prevalence of three-generation households at the group level is a proxy for access to intergenerational support within that group (cf. Reher 1998), and less affected by endogeneity. Conditioning three-generation household prevalence on grandparental presence in Sweden excludes variation due to policy or migration history reasons which may restrict the possibility of forming such households and tap more closely to willingness for closer kin support.

Because of our small number of ancestry groups, we did not perform a multilevel analysis, but instead visually inspected and correlated the regression coefficient estimates with the aggregate measures, akin to what is sometimes referred to as a “two-step analysis” (Bowers and Drake 2005; Bryan and Jenkins 2016).

## Results

**Fig. 1:**
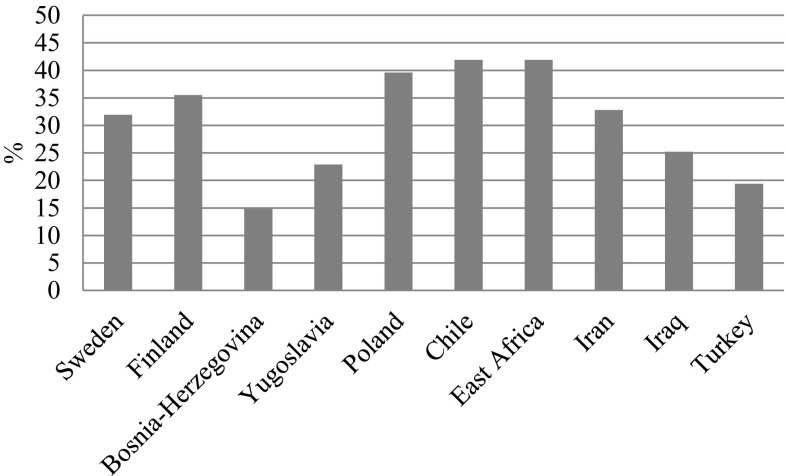
Parental separation by age 15, by ancestry (%)

Figure 1 displays the share of children who experienced parental separation by ancestry group. Parental separation incidence varies considerably. Fifteen percentage of children with Bosnian-Herzegovinian mothers experienced parental separation, compared to about 40% or more of those with Polish, Chilean, or East African mothers. Children with Swedish-born parents are found in between, with roughly one-third experiencing parental separation by the time their grades are measured.

Table 2 (above) provides further comparisons between the groups in terms of the other independent variables. The groups show rather major differences. Large differences are found in the parents’ educational levels. Only 10% of Turkish-origin mothers, but up to 45% of mothers from Iran had post-secondary education. For most groups, the educational levels of the mothers in our sample correspond to those of all women from these origin countries (in 1995, not shown). However, the mothers born in Finland and Iraq are more educated than the average woman from their origin country, whereas the opposite is true for mothers from Chile and Turkey. Although mothers’ employment rates were high in all groups, they ranged from 70% among Iraqis and East Africans to 94% among Swedish-born mothers, and similar differences can be found in household disposable income levels. The year of arrival to Sweden reflects the groups’ migration histories. Finnish mothers had, on average, arrived as children in the 1970s, whereas the average Bosnian-Herzegovinian, East African, Iranian, or Iraqi mother arrived in the 1990s. The mean number of children born to mothers ranges from 2.3 among Iranian mothers to 4.6 among East Africans.

Table 3 shows the parental separation gap in grade sums and the prevalence of failing one or more core subjects by ancestry. Also the parental separation gaps show major variation, ranging from 16 to 17 grade sum points among children with East African or Chilean ancestry to 33 grade sum points among those with Bosnian-Herzegovinian mothers. The latter gap is over half of the standard deviation and 1.4 times the grade sum gender gap (23 points). Likewise, the parental separation gap in receiving one or more non-passing grades ranges from 3.6 percentage points (OR 1.3) among those with Chilean mothers to 12.4 percentage points (OR 3.3) among youths with Bosnian-Herzegovinian mothers. Both gaps are of similar size among youths with Swedish mothers and youths with Finnish, Yugoslav former Yugoslavian, and Polish mothers. Otherwise, the patterning of the crude gaps escapes any simple categorizations. When parental separation is uncommon, the gaps are large in one group (Bosnia-Herzegovinian mothers) and small in another (Turkish mothers), and small (Chile) to average (Poland) in groups where parental separation is common. Neither do the patterns clearly cluster according to the groups’ migration histories nor along geographical or religious-cultural lines.

Do these patterns remain when we adjust for the control variables, and can they be explained by the mediating variables? Table 4 presents the results from the regression models run separately for each included  ancestry group (the estimates of the control and mediating variables are not presented but available upon request).

**Table 4:** Regression analysis of grade sums (ordinary least squares) and incomplete grades (linear probability models) by ancestry

	Grade sum (OLS regression)		Incomplete grades (LPM)	
	Model 1	Model 2	Model 1	Model 2
Bosnia-Herzegovina	−28.0***	−23.4***	0.103***	0.079**
Chile	−10.8*	−4.9	−0.003	−0.025
East Africa	−13.8**	−9.1*	0.063**	0.041
Finland	−18.6***	−14.5***	0.039**	0.028*
Iran	−16.8***	−12.8**	0.062**	0.056**
Iraq	−16.4***	−13.7**	0.036	0.031
Poland	−22.6***	−20.3***	0.054*	0.045*
Sweden	−17.9***	−13.8***	0.045***	0.035***
Turkey	−19.9***	−16.4***	0.077**	0.066*
Yugoslavia	−24.2***	−18.3***	0.092***	0.066***

Model 1 controls for gender, birth year, mother’s and father’s education, mother’s age at birth of the child (linear and squared), number of siblings (mother’s side), birth order (mother’s side), birth municipality, time since immigration, and whether the father was born abroad; Model 2 additionally controls for logged disposable incomes and mother’s employment status

* *p* < 0.05; ** *p* < 0.01; *** *p* < 0.001

Model 1 presents results from the model that adds the control variables (gender, cohort, education of both parents, mother’s age at birth (linear and squared), family size, birth order, and whether the father was an immigrant). Introducing the control variables reduces the parental separation gap in grade sums (first column from the left) as well as in incomplete grades (third column from the left) in each group except for children with Turkish-origin mothers.

When looking at grade sums, these control variables explain approximately one-third of the parental separation gap for children with mothers from Sweden, Finland, and Chile, around one-fifth of the gap for children with mothers from Bosnia-Herzegovina, East Africa, Iran, Iraq, and Poland, and approximately one-eighth of the gap for those with Yugoslavian mothers. In other words, parental separation tends to be a common experience among groups experiencing disadvantage (in terms of school performance), in descending order of relative disadvantage. Youths with Turkish-origin mothers are the exception: In this group, parental separation is a more common experience among better-performing youths and the gap conditional on the control variables is larger than the crude gap. The results are generally similar when incomplete grades are used as the dependent variable (although they explain more of the gap for children with mothers born in Chile or Iraq). These findings show that selection by the background variables is of varying importance. The negative selection effect tends to be strongest for children of Swedish, Finnish, and Chilean descent, weakest for children of Yugoslavian descent, and the reverse for children of Turkish descent.

Model 2 adds the mediating variables mother’s employment and (logged) disposable incomes. The decreasing coefficient estimates suggest that these variables further explain the parental separation gap in school grades. The gap measured in grade sums is cut by over half and becomes nonsignificant for youths with Chilean parents and is reduced typically by around 15–30% in the other groups. Similar reductions are witnessed in the gap measured as incomplete grades, which is no longer statistically significant for youths with East African mothers. The effects of parental separation thus operate partly through its socioeconomic consequences.

The remaining net parental separation penalty—regardless of the outcome measure—is the largest for youths with Bosnian-Herzegovinian mothers, 23 grade points (i.e., approximately 1/3 standard deviation, or equal to the gender gap in grade sum) or 7.9 percentage points for receiving non-passing grades. The net penalties are the smallest—not statistically significantly different from zero—for the Chilean ancestry group; for non-passing grades, they are not significant also in the Iraqi and East African groups. We proceeded with a more detailed analysis of these group differences. First, we estimated the full interaction models to directly compare the penalties between the groups and how they change with the inclusion of additional variables.

Table 5 shows the results from the interaction models. Bosnia-Herzegovina, which also had the largest parental separation gap, is used as the reference group. Model 0 presents the estimates from a model without control or mediating variables. The parental separation gaps in grade sums are smaller (interaction coefficients are positive) and statistically significant in the Chilean, East African, Iraqi, and Turkish ancestry groups. For non-passing grades, the gap is additionally significant in comparison with Finns. After adding the confounding variables (Model 1), the grade sum gap between Bosnian-Herzegovinian and Turkish groups disappears, whereas the differences between the former and those with Swedish and Finnish mothers increase and become statistically significant. The gap in non-passing grades between Bosnian-Herzegovinians and East Africans and Turks also becomes nonsignificant. These results repeat the findings discussed above: the smaller crude gap among children with Turkish-born mothers is partly due to the small socioeconomic differences between dissolved and intact families, whereas low socioeconomic status is a stronger predictor of family dissolution among Swedish and Finnish than among Bosnia-Herzegovinian families, which translates into larger crude gaps in the former groups.

**Table 5:** Full interaction models between ancestry and the independent variables, OLS regression (grade sums) and logistic regression (incomplete grades)

	Grade sum (OLS)			Incomplete grade (logistic, OR)		
	Model 0	Model 1	Model 2	Model 0	Model 1	Model 2
Par. separation (Ref. Bosnia-Herz.)	−32.8***	−28.1***	−23.5***	3.34***	2.81***	2.10***
Chile	16.1*	18.3**	20.5**	0.38**	0.35**	0.39**
East Africa	16.7**	14.8**	14.7**	0.52*	0.61	0.66
Finland	5.9	9.7*	9.3	0.57*	0.57*	0.64
Iran	9.3	11.5	10.8	0.77	0.90	1.04
Iraq	11.9*	11.7 *	9.8	0.47**	0.50*	0.62
Poland	4.1	5.5	3.3	0.77	0.73	0.85
Sweden	5.9	10.3*	9.7*	0.72	0.67	0.76
Turkey	14.3*	8.2	7.4	0.48**	0.63	0.74
Yugoslavia	5.0	3.4	4.7	0.66	0.73	0.76

Model 0 is without control variables; Model 1 controls for gender, birth year, mother’s and father’s education, mother’s age at birth of the child (linear and squared), number of siblings (mother’s side), birth order (mother’s side), birth municipality, time since immigration, and whether the father was born abroad; Model 2 additionally controls for logged disposable incomes and mother’s employment status. Ancestry interacted with all independent variables

* *p* < 0.05; ** *p* < 0.01; *** *p* < 0.001

Adding the mediating variables (Model 2) makes the estimates for Finns and Iraqis nonsignificant; the smaller economic penalties of parental separation in these groups explain part of the differences.

**Fig. 2:**
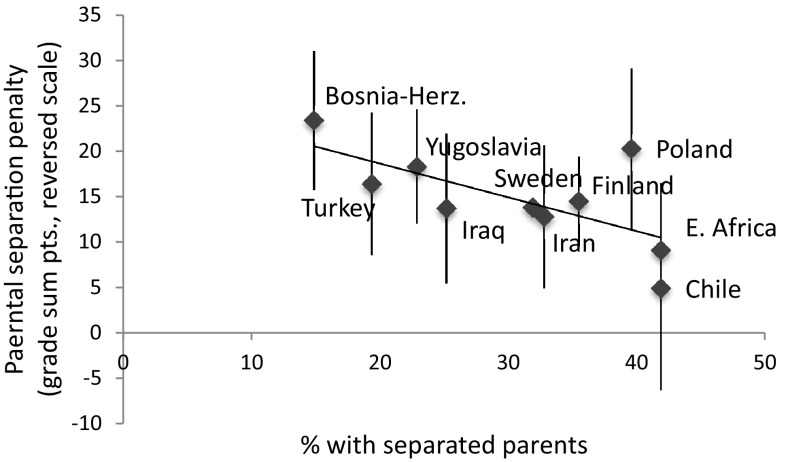
Parental separation occurrence and parental separation penalty (in GPA pts, from model 2, Table 3). Corr = −0.67

**Fig. 3:**
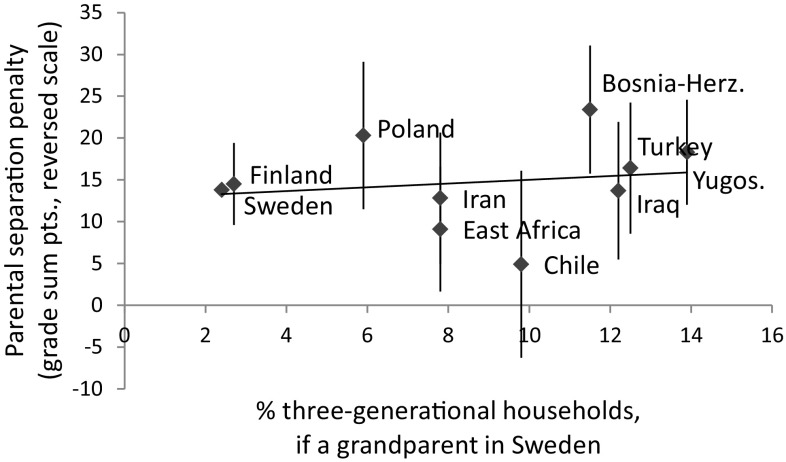
Prevalence of three-generation households and parental separation penalty (in GPA pts, from model 2, Table 3). Corr = 0.17

Finally, we inspect the association between the remaining parental separation penalty (coefficients from Model 2) for grade sums with the share of youths who experienced a parental separation as well as the prevalence of three-generation households (conditional on grandparental residence in Sweden). Our findings are shown in Figs.  2 and 3. Figure 2 shows that the net parental separation penalty in grade sums is weaker in ancestry groups in which parental separation is a more common experience (correlation −0.67). The relationship is robust to outliers. This result is in line with Kalmijn’s (forthcoming) analysis, who  found a similar pattern for father absence and youth’s well-being, and more generally with the argument of weaker parental separation penalties when parental separations are a more common experience. On the other hand, there is no clear relationship between the prevalence of three-generation households and the net parental separation penalty on grade sums (correlation 0.17). Due to the limitations of this measure of kin support, we cannot disprove the hypothesis that the parental separation penalty is weaker in groups where the presence of kin (or other social support) is stronger. However, neither does this finding provide support for this argument (Hypothesis 4).

## Discussion

We analyzed whether the parental separation gap in school grades (measured at the end of the ninth grade of comprehensive school) varies between ancestry groups (measured by the country/region of birth of the mother) in Sweden. We compared ten groups: youths with mothers born in Sweden, Bosnia and Herzegovina, Chile, East Africa (Djibouti, Eritrea, Somalia, Sudan, and Ethiopia), Finland, Iran, Iraq, Poland, former Yugoslavia (excluding Bosnia and Herzegovina), and Turkey. These represent large immigrant groups in Sweden and the heterogeneity of its immigrant population in terms of geographical, cultural, and economic developmental origin and integration in Swedish society. Our study is among the first outside the USA to compare parental separation penalties across minority groups (for previous analyses, Kalmijn 2010, forthcoming). Furthermore, our study contributes to the research on heterogeneity in parental separation effects more generally, a topic which has received increasing attention among researchers (Amato 2010).

We documented rather considerable heterogeneity in crude parental separation gaps. These ranged from 16 to 33 grade sum points (corresponding to ¼ to ½ of a standard deviation, or ¾ to 1½ gender gaps) and from an odds ratio of 1.3–3.3 in having incomplete grades, which disable progression to standard secondary school programs. The raw gaps were in many cases considerably reduced (and often lost statistical significance) when controlling for social and demographic background and the mediating variables maternal employment and household disposable income. The remaining net parental separation penalties in grade sums ranged from not significant (Chilean background) to 23 (Bosnian-Herzegovinian ancestry), and in incomplete grades from not significant (Chile, East Africa, Iraq) to 7–8 percentage points (Bosnia-Herzegovina, the rest of former Yugoslavia, and Turkey). Penalties for youths with Swedish-born mothers were in between.

In terms of explaining the between-group variation in parental separation gaps, our analysis highlighted some important findings. First, we found that socioeconomic and demographic variables explained some of the differences between groups. Second, our analysis also showed that the confounding and mediating effects of these variables can differ between groups. In some groups, such as among those with native Swedish mothers, these variables explained up to half of the raw association between parental separation and school performance. In these groups, children of divorce come from more disadvantaged backgrounds, such as families with low education. In other groups, such as those with Turkish mothers, the socioeconomic and demographic background variables did not explain the grade gap. Quite the opposite, the parental separation gap even increased when these variables were adjusted for.

Second, our results gave support for the argument that parental separation penalties are smaller in groups where parental separation is more common (Amato and Keith 1991; McLanahan and Sandefur 1994; Kalmijn 2010; forthcoming). The common explanation for this finding is that the effects of family dissolution are weaker when family dissolution is  more accepted and less stigmatized, when single parenthood is a more institutionalized living arrangement, and when parents and others in the community have better skills to handle family dissolutions. The findings in support of this argument would thus provide an important clue for  understanding heterogeneous family dissolution effects more generally (Amato 2010). However, we questioned the applicability of this interpretation in a society such as Sweden, where disapproval of parental separations is, overall, low (Rijken and Liefbroer 2012). Likewise, findings of stronger parental separation effects in countries with higher rates of family dissolution (Pong et al. 2003; Kreidl et al. 2017) suggest that the “more common, less harmful” argument is not straightforward. As an alternative explanation, we suggested that dissolved families are differently selected (by unobserved factors, such as parental conflict) in communities in which family dissolution is more common. Future research would do well to assess more direct measures of stigma and other factors deemed as important mechanisms behind this association. Our analysis did not support our hypothesis of weaker effects in groups in which kin support can be more accessible—proxied by the prevalence of three-generation households; however, the limitations of the measure we used have to be considered.

Our findings have implications for research on immigration and inequality. As reported in the descriptive analysis, the likelihood of experiencing parental separation varies considerably between ancestry groups in Sweden. Whereas 15% of the children of mothers born in Bosnia-Herzegovina experienced parental separation, the share among children of East African, Chilean-, or Polish-born mothers was around 40%. For comparison, the respective figure for those of Swedish-born mothers was just above 30%. To the extent that parental separation leads to poorer school performance, the differences in parental separation incidence (and other family demographic events) can strengthen inequalities among children of different ancestries. On the other hand, the generally weaker parental separation penalties in groups in which parental separation is more common works to counteract this unequalizing effect in ways similar to weaker parental separation penalties in low-SES families, reported in some studies (Bernardi and Boertien 2017, this issue). In this respect, groups with both a high occurrence of parental separation and strong parental separation penalties (here, children of Polish mothers) can experience a double penalty.

Future research should build on these analyses in Sweden and elsewhere in order to better understand the role of family dynamics in shaping inequality among ancestry groups as well as for understanding the heterogeneity in parental separation effects. Some suggestions can be given. First, reproducing these results for the same immigrant groups in other countries is welcome. Not only can the features of the host country shape these effects, but they can also be shaped by differential selection from the same country of origin to different countries of destination, as well as differential selection in staying in that country. Future research could also consider parental separation effects explicitly among children of mixed ancestry (cf. Panico and Nazroo 2011; Platt 2012). Second, the question of causality of effects haunts all research in this field (e.g., Härkönen et al. 2017, this issue) and can be addressed within research on group differences in parental separation as well. Third, richer data with direct measures of the hypothesized mechanisms (such as social support) that relate parental separation to school performance can be useful, as can data with more specific information on immigrants’ migration experiences. Here, for example, we could not assess whether the immigrants had arrived in Sweden as refugees, although the variation in the penalties between groups, which included many refugees in the 1990s (e.g., Bosnia-Herzegovinians, East Africans, and Iraqis), suggests this omission would not explain group differences. Fourth, continued research would likewise do well to consider multiple family forms and dynamics. Our finding of variation in gaps among children from intact families, single mothers, and dissolved families suggests that family dynamics other than separation can vary between groups (also, cf. Kalmijn forthcoming).

Research building on these findings should also address different educational outcome measures. Our outcome measures were general measures of school performance, and even though they are good predictors of later educational attainment, it is possible that variation in parental separation effects would manifest more strongly in specific subjects, such as Swedish. Previous studies have found that although many immigrant groups show weaker performance in terms of grades, they have a higher likelihood of pursuing educational tracks that lead to university (Jonsson and Rudolphi 2011). Whether some groups manage to compensate for parental separation penalties in grades by higher probabilities of choosing advanced educational tracks is among the exciting topics for future research.
